# Impacts of climate change on household food security in Matande communal lands, Mwenezi district in Zimbabwe

**DOI:** 10.4102/jamba.v15i1.1499

**Published:** 2023-09-21

**Authors:** Fanuel Muzerengi, Crecentia P. Gandidzanwa, Lovemore Chirubvu

**Affiliations:** 1 Department of Community and Social Development, Faculty of Social and Behavioural Sciences, University of Zimbabwe, Harare, Zimbabwe

**Keywords:** adaptation, climate change, food security, practices, household, vulnerability

## Abstract

Mwenezi district is a drought prone area characterised by high temperatures, droughts, rainfall deficit, crop failures and chronic food deficiencies. Rainfed agriculture can no longer be sustained without any innovations. The study explored the impacts of climate change on household food security among the vulnerable populations of Matande communal lands, Mwenezi district in Zimbabwe. The study is guided by the Sustainable Livelihoods Framework (SLF). An exploratory sequential research design was adopted, and a total of 78 respondents were selected from the population of 371 households using purposive and cluster sampling techniques. Data collection was triangulated through the use of household survey, focus group discussions, key informant interviews and observations. The thematic approach and SPSS software were used to analyse qualitative and quantitative data, respectively. Results demonstrated that climate change propelled increases of pests and diseases for both livestock and crops, reduction of meals uptake per day, biodiversity loss and dwindled crop production. Livestock increases were recorded despite the changing climate shows a nexus with food insecurity. The research called for the adoption of drought-tolerant crops, capacity building through climate change resilience programmes, livestock centric in diversification, improved formal markets for livestock and tapping of underground water for irrigation and other purposes to complement existing water bodies to prevent them drying up early.

**Contribution:**

There is deepening aridification in Mwenezi district because of climate change resulting in the continuous obliteration for the worst of agro-ecological regions iv and v reclassified into a and b. This confirmed the heterogeneity of various climatic conditions and variability within the same geographical context. However, vulnerability continues to be generalised into regions. The study investigates the impacts of climate change typical to Matande communal lands with the view to generate knowledge relevant to review adaptation practices specific to the researched area in order to escalate community resilience.

## Introduction

The effects of climate change are ever growing and tremendously increasing vulnerability to all the populations of the world (Sibanda, Matsa & Maswoswere 2021). Related disasters have escalated globally, posing a serious threat to humanity and their livelihoods (Mugambiwa 2018; Tirivangasi 2018). The negative consequences are dominating (Ding et al. 2021; Food and Agriculture Organization [FAO] 2015; Intergovernmental Panel on Climate Change [IPCC] 2018; Muzerengi & Tirivangasi 2019). Global food security statistics projected that after the coronavirus disease 2019 (COVID-19) disaster, the next one is likely to be a hunger pandemic (World Food Programme [WFP] 2021). In 2019, a total of 700 million people were food insecure globally, followed by 832 million people in 2020 (WFP 2021), and the figures projection will skyrocket to 1014 billion by the year 2050 (IPCC 2018). Around 80% of the world’s population is most at danger from crop failures and famine brought on by climate change (FAO 2016). Saliya and Wickrama (2021) predicted that millions more people could fall into poverty as a result of a severe drought brought on by the El Nino weather pattern or climate change.

The terms food security and food insecurity have been used interchangeably although they mean different things. The World Food Summit (1996:17) defined food security as, ‘access to safe, nutritious and sufficient food which meets dietary needs and food preferences for an active life and healthy life’. This study conceptualised food security as denoting production, availability, access to nutritious and stability in food supplies over time. On the contrary, FAO (2016:33) described ‘food insecurity’ as, ‘a situation that exists when people lack secure access to sufficient amounts of safe and nutritious food for normal growth and development, and an active and healthy life’. ‘Food insecurity’ thus can be best seen as a condition of lack of access, improper utilisation, non-availability of food and instability for a period. Because of food insecurity, at-risk populations become vulnerable.

Vulnerability can be understood as the susceptibility of individuals, households or communities to the negative effects of climate change and other factors that compromise their ability to access and maintain an adequate and nutritious food supply (FAO 2021). On the contrary, the United Nations Office for Disaster Risk Reduction (UNISDR) (UNISDR Terminology 2017) defined vulnerability as ‘the characteristics and circumstances of a community, system or asset that make it susceptible to the damaging effects of a hazard’. It is considered that vulnerability is being susceptible to adverse conditions. However, Filho and Nalau (2018) argued that the degree of vulnerability is not homogenous because of gender and resources among other things.

The issue of climate change is regarded as a question of depletion, disappearance and collapse of the ecosystems, economies and food systems because it has brought more harm than the good in many societies (Elliot 2018; Filho & Nalau 2018). The world is being haunted by the effects of industrial, transport and agricultural revolutions happened in Europe that triggered climate disruptions witnessed today (Coppola et al. 2021). They generated greenhouse gases that continued to accumulate. What is disappointing arguably is that the effects of climate change are disproportionately felt in the global South who are least able to mitigate the harms despite having contributed less to it (Filho & Nalau 2018; Gukurume 2013; Maino & Emrullahu 2022; Williamson et al. 2020). Europe is the least affected continent because it has resources to adapt and insulate against the impacts of climate change (Burke & Lobell 2010; IPCC 2018). On the contrary, IPCC (2018) attested to the fact that Africa has almost done nothing to the problem of climate change by contributing 3% of the global emissions but is the one suffering the most. The African Development Bank (2022) attested to the fact that the continent is reportedly loosing up to $15 billion per annum because of climate change, and if financing is not availed any time soon, the losses will skyrocket to $50bn yearly.

Drought and food insecurity are closely related. Drought, characterised by prolonged periods of inadequate precipitation, severely affects agricultural production and can lead to crop failure, livestock loss and depletion of water sources (IPCC 2014). Drought reduces the availability of water for irrigation, limits crop growth and decreases livestock productivity. As a result, households relying on rain-fed agriculture face decreased food production, income loss and heightened food insecurity. Drought exacerbates existing vulnerabilities and can push already vulnerable populations further into food insecurity (WFP 2020). The above definitions were operationalised for the purposes of this study.

Betran-Pena and D’ Odorico (2022) and Mavhura, Manatsa & Matiashe (2017) extrapolated that Africa is a major hotspot for food insecurity and climate change vulnerability because most countries in Africa are not currently and will not be self-sufficient under a changing climate. Nine out of 10 African nations are extremely susceptible to food insecurity because of climate change (The African Development Bank 2022; The Global Centre for Adaptation 2022). Africa’s economy is hinged on rain-fed agriculture such that when agriculture is affected, its economies had problems too (Chanza 2018; Filho & Nalau 2018). Matunhu (2012) concurred and stated that increased levels of poverty in Africa have forced people to live under vulnerable conditions. In 2018, Ethiopia, Malawi, Kenya, Mozambique, Madagascar, Zambia and Uganda were the most affected by climate shocks from adverse weather conditions, drought, floods, late or erratic rainfall (FAO 2020). In the Lake Chad region, temperance over scanty water resources has become an instrument of war for both abhorrent and defensive aims among locals (Cappelli et al. 2022).

Climate change burdens the already at-risk populations (Alam 2016, 2017; Brown et al. 2012; Chanza 2018; Zimbabwe Human Development Report 2017). Zimbabwe is no exception. Vulnerability to climate change is classified from natural farming region i to region v (Bhatasara 2017). The differentiation is according to average rainfall, soil type, temperatures, altitude and vegetation type (Bhatasara 2017; Chikodzi et al. 2013; Chitongo et al. 2019; Nyikahadzoi & Mhlanga 2021). The regions’ capacity to produce diminished as they are going up. Food insecurity in the study area is further compounded by its geographical location. The Green Climate Fund (2019) stipulated that 90% of Mwenezi district falls under natural farming region v characterised by serial droughts, poor soils, erratic precipitation, hit waves and cyclones.

There is also increased aridification in Mwenezi district because of climate change in which agro-ecological regions iv and v were further reclassified into a and b (Chikodzi et al. 2013). The continuous obliteration of agro-ecological regions for the worst lead to increased aridification (Chikodzi et al. 2013). The Zimbabwe Nation Climate Change Response (Government of Zimbabwe 2017) predicted that by 2080, excellent maize growing area will be reduced from 75% to 55%. Heat waves make food less nutritious (WFP 2021). The food security situation is precarious in Mwenezi district; maize, which is the staple crop in the area, cannot tolerate abiotic conditions in the area (Muchara 2010). Hunger, malnutrition, stunting and starvation have notoriously become residents in Mwenezi district. International Fund for Agricultural Development (IFAD 2020) and The Green Climate Fund (2019) have persistently classified Mwenezi district in emergency category implying that its people are persistently food insecure. In 2018 alone, an estimated 72 872 people in Mwenezi district were food insecure (Zim VAC 2019). Zim VAC (2022) further attested to the fact that 69% of households in Mwenezi are perpetually cereal insecure from January to March every year.

Alam (2017) and Chanza (2018) confirmed the heterogeneity of various climatic conditions and variability within the same geographical context. However, vulnerability is generalised into regions, yet the same area has different relief, soil type and variability climate (Chanza 2018; IPCC 2022; Shava & Gunhidzirai 2017). The objectives of the research were to establish the impacts of climate change and household food security among small-scale farmers as well as to develop sustainable adaptation practices to enhance food security in the drought risk zones. To this end, the research investigated the impacts of climate change typical to Matande communal lands with the view to generate knowledge relevant to review or develop adaptation to climate change for sustainable household food security specific to the researched area in order to address perennial food shortages.

## Theoretical framework

The study is guided by the Sustainable Livelihoods Framework (SLF). Sustainable Livelihoods Framework provides a comprehensive theoretical framework for understanding the impacts of climate change on household food security in Matande communal lands. This framework encompasses five key components: livelihood assets, livelihood activities, livelihood strategies, livelihood outcomes and the vulnerability context (DFID 1999). By applying the SLF, this study can examine the diverse resources and assets that households possess (Ellis 2000), their livelihood activities (Chambers & Conway 1992) and the strategies they employ to cope with climate change impacts (Adger 2003; Scoones 1998). It can assess the outcomes of these strategies on household well-being, particularly in terms of food security (Carney 1998). Furthermore, the SLF allows for an analysis of the broader vulnerability context, including social, economic, political and environmental factors that shape households’ vulnerability to climate change (DFID 1999). Overall, this framework offers a holistic approach to understanding the complex dynamics between climate change, livelihoods and food security, enabling the identification of appropriate interventions and policy recommendations to enhance resilience and sustainable livelihoods in Matande communal lands.

## Research methods and design

### Study area

**FIGURE 1:**
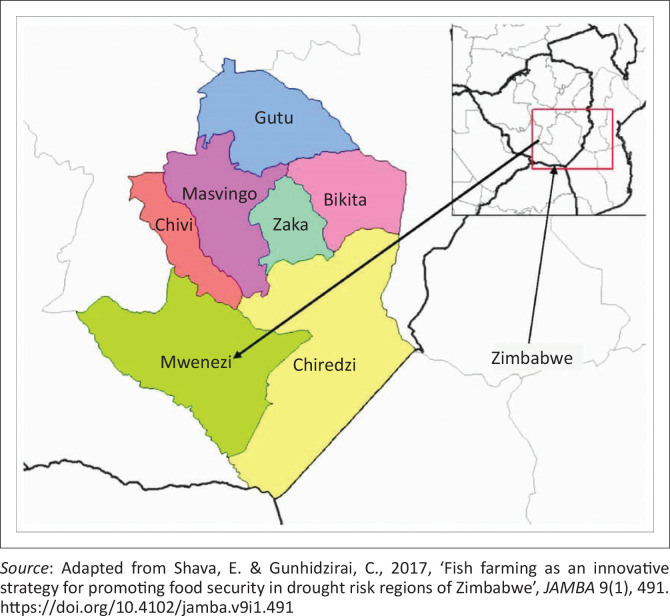
Location of Mwenezi District, Zimbabwe.

The investigation focused on the impacts of climate change in Matande communal lands, Ward 2, Mwenezi district in the southeast of Zimbabwe (see Figure 1). The district is situated 120 km to the south of the provincial capital Masvingo (Mutopo 2014). The district shares borders with Chivi to the north, Chiredzi to the east, Bubi to the south and Mberengwa district to the west. The area of the study consisted of Matande (154 households), Rutavo (157 households) and Mashindi (60 households) villages. The drainage system of Matande communal lands is dominated by Runde, Shashe, Chivake and Mamwa Rivers. The district was purposively selected for the study for being a drought risk zone where there is evidence of the adverse impacts of climate change. The district is situated partly in the agro-ecological regions iv and v, which had the harshest climatic conditions in Zimbabwe (Muchara 2010). It received an average rainfall of 540 mm per year and highly abnormal temperatures above 25°c and low temperature of between 10°C and 15°C in the hot summer period (Brazier 2015; Shava & Gunhidzirai 2017). Rain-fed agriculture has become a risk venture as such the study area has been perpetually food insecure because of climate change.

The area has Savannah type of vegetation and dominated with sand soils. Livelihoods are largely agrarian with maize as a staple crop. Farmers rely on subsistence livestock rearing; growing small grains, legumes, mbambara nuts; and migration into South Africa (Mutopo 2012; Shava & Gunhidzirai 2017; Zim VAC 2022). The majority of households are pesistently food insecure when it comes to cereals as evidenced by statistics. The Zim VAC (2022) attested to the fact that 69% of households in Mwenezi were cereal insecure and food insecure. Before the Fast Track Land Reform Programme (FTLRP) in 2000, the majority of the area was reserved for cattle ranching (Frischen et al. 2020).

### Research methods

Yin (2003:19) averred that colloquially, a research design is an action plan for getting from here to there, where ‘here’ may be defined as the initial set of questions to be answered, and ‘there’ is some set of (conclusions) answers. The research utilised an exploratory sequential research design in which qualitative and quantitative data were collected and analysed. It builds on sequential timing in which qualitative results inform the quantitative designing (Creswell & Plano Clark 2011, 2018; Enosh, Tzafrir & Stovolovy 2015). The qualitative methods were used to elucidate the lived experiences and households’ perception, while the quantitative ones collected numerical data on the effects of climate change on food security.

### Population and sample size

Population refers to the totality of subjects under study (Acharya et al. 2013). This study has a total of 371 households in Matande communal lands. The study was not sponsored; hence, it was not feasible to collect data from individual members of the study population and proceeded with sampling.

The researchers used purposive sampling and cluster sampling to identify 78 study participants. Respondents for the qualitative study were determined by saturation of data (Kumar 2011), which was reached at 33 respondents. Sampling for the quantitative study was determined by the rule of the thump by Cohen, Manion and Morrison (2007:101) who enunciated that a household survey research sample must have at least 30 participants. The researchers increased the sample size in quantitative research from the recommended minimum of 30 to 45 respondents to deal with poor response rate (Punch 2003:43).

Through purposive sampling, researchers recruited household heads who were elderly and resident in Matande communal lands for the past 10 years who had experienced and were able to make a comparison with yesteryears on the impacts of climate change on household food security. Denzin and Lincoln (2005), Kumar (2011:17) highlighted that ‘… in qualitative research, data is usually collected to a point where researchers are not getting new information or it is negligible’. The saturation point of qualitative study consisted of 9 key informants and 24 small-scale farmers. Cohen et al. (2007) and Ball (1990) argued that purposive sampling is utilised with the aim of getting ‘knowledgeable people’ possibly because of their professional part, power and access to networks or skill. Purposive sampling was for expert identification and critical case sampling from government departments, traditional leadership and non-governmental organisations operating in the area.

This study utilised cluster sampling to identify 45 respondents for a household survey. Matande communal lands was already clustered into three villages, namely, Matande (154 households), Rutavo (157 households) and Mashindi (60 households); hence, it was easier to cluster respondents. Cohen et al. (2007:112) postulated that in cluster sampling, it is safer to take several clusters and to sample lightly within each cluster, rather to take fewer clusters and sample heavily within each to ensure representativeness. The study purposively identified three sub-clusters from every village which consisted of respondents each from the centre, far west and far east of each of villages to ensure that clusters do not build biases.

### Data collection and analysis

The researchers triangulated the data collection tools in which in-depth interviews, key informant interviews, FGDs, observation and a questionnaire were used. Triangulation is defined as a process of using multiple perceptions to clarify meaning, verifying the repeatability of an observation or interpretation (Denzin & Lincoln 2008:133). All unstructured interviews done by the researchers followed the progression of questioning as prescribed by Kvale (1996:133–135), Berg (2001:70), Johnson and Christensen (2008:207) and Turner (2010:58; Silverman 2010).

The researchers conducted two FGDs with elderly community members who were resident in the study area for the past 10 years to get their lived experienced on the effects of climate change on household food security. The FGDs comprised of 10 respondents for each group, four men and six women to ensure representativeness of the population. Climate change affects men and women within agrarian populations differently (Denton 2002; Ngum & Bastiaensen 2018). More women were included because they were readily available, and the majority of men had migrated from the rural areas to search for employment. The researchers started by mining data using FDGs in order to get diverse information and cover much groundwork on climate change impacts while acquiring diverse knowledge on the subject.

In-depth interviews were used after FGDs. A total of four in-depth interviews were conducted on three lead small-scale farmers and one household head living with disabilities to understand their perceptions and how they were personally affected by climate change over the test of time in Matande communal lands. Respondents were adequate because qualitative research is premised on a small sample in order to get in-depth knowledge on the researched topic (Denzin & Lincoln 2005).

Key informant interviews involved the intense oral individual questioning of technical persons (Bryman 2008:192; Green & Thorogood 2009:94; Keyton 2001). Researchers handpicked AGRITEX officer (1), Veterinary officer (1), Meteorologist (1), District development official (1), Social Welfare officer (1), Village head (1) and Non-governmental organisation (3), which are World Vision, CARE International and World Food Programme. The researchers concluded unstructured interviews by using key informant interviews to enable technical persons to tie loose ends raised in the former data mining instruments (Denzin & Lincoln 2005) on impacts of climate change on household food security.

The researchers conducted the study when small-scale farmers in the researched area were doing land preparations and planting for the 2022/2023 planting season. They observed the activities on climate change adaptation strategies as they were being executed, mal-adaptation practices and the impacts of climate change were visible in Matande communal lands. Silverman (2010:222) posited that ‘practically all senses- seeing, hearing, feeling and smelling are integrated into observation’. The researchers go beyond gathering data from people who do not want to take part in the enquiry (Silverman 2010).

Lastly, questionnaires were used to obtain quantitative data. Cohen et al. (2007) and Creswell and Clark (2018) defined questionnaire as a research instrument made up of a set of questions for the objective collection of data for a statistical survey. Questionnaires were administered on small-scale farmers to establish changes brought on by climate change and determine if those changes had a positive or negative impact on the respondents’ livelihoods. Questionnaires were distributed face to face and strategically to respondents from the centre, far west and far east of the three clusters identified to ensure the reliability of data. They were collected after two days so that respondents are not pressured and have ample time to answer questions earnestly. Out of 45 questionnaires distributed, two were not returned by respondents citing that they had misplaced. Through the use of a questionnaire, standardised responses were obtained (Enosh et al. 2015).

Matande communal lands is highly patriarchal; study participants who were women sought spousal validations in order to participate in the study, and hence, the research needed more time. The researchers had to wait for their decision and proceeded with the study.

### Data analysis

The analysis for qualitative data was done using the thematic approach. Braun and Clarke (2006) saw thematic way as systematically identifying, organising and proposing insight into patterns of meaning (themes) across a data set. The researchers, followed a six-phased process, began with being familiar with the data, generating initial codes, searching for themes, reviewing themes, defining and naming themes, and finally producing the report as espoused by Braun and Clarke (2006). Statistical analysis was done for quantitative data in Statistical Packages for Social Scientists (SPSS) software using cross-tabulations and graphs. In order to determine the impacts of climate change and food security at the household level, binary responses (1-Yes, 2-No) from questionnaire data were entered and transformed into a contingency table. The contingency table showed data responses on the impacts of climate change on household food security status against the corresponding effect.

### Ethical consideration

Kvale (1996), Kombo and Tromp (2006), Cohen et al. (2007), Silverman (2010), Pirot and Beck (2012:156), and Creswell and Creswell (2018) defined ethics as the conduct of the investigator that is proper in relation to the rights of those subjects under the study or those affected by it. The researchers sought informed consent prior to data mining from each respondent. The study upheld the right to privacy through coding data, and the respondents were allowed to withdraw at any stage during fieldwork such that they thanked those who even misplaced questionnaires. Researchers also have a clearance letter from the University of Zimbabwe to carry out the study. Ethical clearance to conduct this study was obtained from the University of Zimbabwe, Department of Community and Social Development.

## Results

### Response rate from questionnaires

Table 1 indicates the rate of response of the questionnaires administered to respondents. Results on Table 1 show that 45 questionnaires were administered on respondents to mine quantitative data. Forty-three questionnaires were completed and returned. Two respondents did not return them citing that they had misplaced them and withdrew from the study. The response rate stood at 96%, which is well above the 60% recommended by Mugenda and Mugenda (2003).

**TABLE 1:** Response rate from questionnaires.

Questionnaires administered	Questionnaires returned	Questionnaires not returned	Rate of response
45	43	2	96%

### Demographic characteristics

Table 2 show the socio-demographic information of study respondents. These included gender, age, marital status, occupation, education and religion of the study respondents.

**TABLE 2:** Socio-economic demographic profile for study respondents (*n* = 43).

Variable	Category	Frequency	Percentage
Gender	Male	27	62.8
	Female	16	37.2
Age	15–25 years	2	4.7
	25–35 years	4	9.3
	35–45 years	19	44.2
	45–55 years	4	9.3
	55 + years	14	32.6
Marital status	Single	1	2.3
	Married	30	69.8
	Divorced	4	9.3
	Widowed	8	18.6
Occupation	Semi-skilled	1	2.3
	Professional	11	25.6
	Manual	29	67.4
	Retired	2	4.7
Education status	No education	8	18.6
	Primary	9	20.9
	Secondary	20	46.5
	Tertiary	6	14.0
Household size	Less than 3 residents	8	18.6
	4–6	27	62.8
	7–9	7	16.3
	>9	1	2.3
Household monthly income	Less than $100	28	65.1
	$101–200	6	14
	$201–300	2	4.7
	$301–400	2	4.7
	$401–500	0	0
	$500+	5	11.6

The study findings show that 62.8% of the respondents were men and 37.2% were women. The quantitative study recruited less numbers of women because Matande communal lands is a patriarchal society where women were unable to make participation without seeking spouses for validations. The majority of age group above 55 years (32.6%) and 35–45 years (44.2%) reside in the rural areas and relied on subsistence agriculture for their living. In terms of marriage status, 69.8% of respondents were married, an indication that the community values the marriage institution. Study respondents (67.4%) depend on manual labour for survival. Manual labour jobs are not highly paid, and hence, the population is in poverty. Many of respondents attained secondary education (46.5%). The high rate of literacy among the population influenced the completion of questionnaires by the study respondents.

### Climate change and household food (in) security among small-scale farmers in the drought risk zones

**FIGURE 2:**
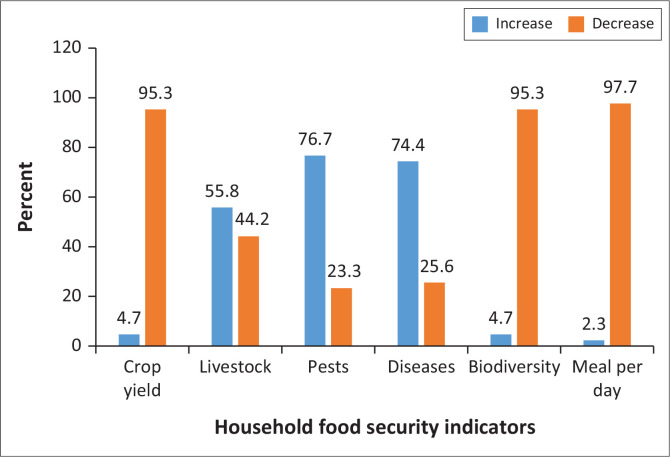
Impacts of climate change on household food security.

Figure 2 shows the domineering negative impacts of climate change on household food security among small-scale farmers in Matande communal lands. Climate change resulted in the reduction of number of meals uptake per day (97.7%), dwindled crop yield (95.3%) and loss of biodiversity (95.3%). On a positive note, the inhabitants of Matande communal lands recorded an increase in the number of livestock (55.8%) at the household level.

Research revealed have dire consequences on household food security. Climate change has resulted in the changing of food consumption patterns such as the reduction in the number of meals per day. During one of the sessions of FGDs, one respondent extrapolates:

‘Now I resolved that we ate one meal per day so that I force my food reserves to take us through to the harvesting time.’ (Widower, 54 years, female, small-scale farmer)

The Riparian ecosystem along Runde River had been altered by environmental degradation caused by flooding and gold panning. On the other hand, droughts negatively affected the production of wild fruits, where some species of fish and wild animals have changed their habitat, thereby compromising food security. A key informant said:

There is a lot of environmental degradation along Runde River mainly caused by gold panning. Look there are pits everywhere as people were searching for gold. Due to low rainfall, the water flow along Runde River was reduced and some seasonal pools have dried up. Tourists and locals used to come here at Chirote to see hippopotamus and sometimes bought tiger fish as well as gathering wild fruits such as makudende along Runde River but now there is none.’ (Government official, environmental, male)

Crops and animal pests as well as diseases wreaked havoc in Ward 2, Mwenezi district because of climate change leading to a highly diminished crop yield. Another small-scale farmer who used solar-powered borehole to irrigate his horticultural plants cited that at one point in November 2022, he had to uproot and burn 8000 plants of tomatoes because they were attacked by an uncontrollable disease. Maize crop was the most affected one bouts of arm worms. One of the interviewed officials reported:

‘Our early assessment of the maize crops planted so far indicated that army worms have already reduced the maize crop by more than half plants per hector.’ (AGRITEX officer)

One respondent also confirmed:

‘Some of us who planted maize with the early rains have realised that we made a huge mistake. A yellow worm is wiping out the maize crops. Now we are planting again. I should have planted small grains; the worms would not have destroyed much of them because they germinated in large numbers.’ (Unemployed, 42 years, small-scale farmer)

There were perpetual crop failures recorded year in and year out because of erratic and often unpredictable rainfall patterns resulting in truncated crop yield. A small-scale farmer where field day commemorations were usually done at his homestead espoused that:

‘I used to have bumper harvests here because of good rains. Look there on the ground, those are marks of a heavy vehicle which got stuck in 2002 when it was loaded with 30 tonnes of maize I have produced and destined for the Grain Marketing Board. Now things have changed, I can no longer able to produce surplus maize, it usually wilted due to prolonged dry spells and heat waves when it is almost mature.’ (68 year-old male, small-scale farmer)

Another confirmed that in the year 2021/2022 farming season, their crop failed and they could not harvest legumes (groundnuts and roundnuts) that are drought resistant because the field had formed a hard crust because of prolonged dry spells:

‘If government failed to provide food aid, by January 2023, we are likely to record death due to food shortages at household level. We had done our homework very well but the weather patterns failed us. I even failed to harvest my ground nuts because the ground was too dry. I just dig a few to get seeds for this season and later quitted.’ (38 year-old, female, small-scale farmer)

Livestock increases were reported despite the negative consequences of climate change. High numbers were found among goats because they are drought tolerant. A general increase was also recorded in the number of cattle in Mwenezi district particularly because of the fact that there was no market for cattle necessitated by sporadic disease outbreaks in the area. People feared that they might buy contaminated meat and get infected. Numbers keep on growing as he indicated:

‘Mwenezi is officially classified under region iv and v which is suitable for animal husbandry.This year, there was no market because the movement of livestock across districts was banned by government to avoid the spread of foot and mouth which was reported in Chivi district. Their numbers keep on increasing day by day. Over the years, our farmers have resisted destocking, they preferred their animals to die during droughts than culling them.’ (Veterinary officer, male, Mwenezi)

The researchers made observations during field work on the impacts of climate change, adaptation and mal-adaptation practices. The mal-adaptation practices witnessed were the rampant cutting down of thorn bushes and indigenous trees for fencing of homesteads, fields and household gardens to protect crops from wild animals, and livestock was causing environmental degradation. The gold panning along Runde River caused siltation and drying up of seasonal pools leading to the migration of animals and marine species. The practice of lending cattle to relatives within the same community to spread the risk was not working, small-scale farmers were recorded colossal loses during droughts as they are often trapped in the same disaster. Mandimo dam that was destroyed by cyclone Eline in 2000 was not repaired. It was observed that the area of the study is mountainous, and there was relief variability in the same area; the western sides of the Dungungwi Mountains were already having crops, while the eastern side of the mountains did not receive any rains by 25 November 2022. The researchers observed that the majority of household small dams used as water harvesting techniques, which were around a few homesteads in Matande communal lands, had dried up.

Table 3 shows the output of raw data transformed from responses in the questionnaires to cross-tabulation using SPSS software version 21 on the impacts of climate change on household food security in Matande communal lands.

**TABLE 3:** Cross-tabulation on impacts of climate change (*n* = 43).

Climate change indicators	Impacts on household food security			
	Increase		Decrease	
	*n*	%	*n*	%
Crop yield	2	4.7	41	95.3
Livestock	19	55.8	24	44.2
Pests	33	76.7	10	23.3
Diseases	32	74.4	11	25.6
Biodiversity	2	4.7	41	95.3
Meal per day	1	2.3	42	97.7

## Discussion

The negatives of climate change were overwhelming in the researched area, which is in agreement with studies by Filho and Nalau (2018), Sibanda et al. (2021) and Chikodzi et al. (2013). Small-scale farmers in Matande communal lands suffered low crop yield because of low, spatial and unpredictable rainfall patterns. Some water bodies such as small dams used by household for irrigation purposes had dried up because of low rainfall and high temperatures. The SLF emphasises the importance of natural resources, such as water bodies and ecosystems, for sustaining livelihoods. The drying up of small dams and decline in freshwater availability, as mentioned above, directly affect the natural capital available to small-scale farmers. These changes reduce their capacity to irrigate crops, impacting agricultural productivity and food security. Matarira, Makadho and Mukahanana-Sangarwe (2004), Chanza (2018), and Zvomuya and Mundau (2021) argued that small-scale farmers in arid and semi-arid regions in Zimbabwe have lost hope in cereal production because of perpetual harvest decline caused by rainfall shortages. There is need for tapping of underground water for irrigation purposes in order to ameliorate climate change impacts in arid and semi-arid regions.

Persistent bouts of army-worm were also found to be retarding crop production and yields. Brazier (2018) and USAID (2016) posited that in the traditionally arid regions in Zimbabwe, army-worm pest can cause crop losses of more than 70% presenting a serious threat to food security. There is need for contingency planning by stakeholders to put in place robust measures to prevent and control crop diseases and pests to ensure sustainable household food security. The SLF emphasises the significance of social networks, institutions and collective action in shaping livelihood outcomes. The need for stakeholders to engage in contingency planning, establish measures for disease and pest control and improve formal livestock markets indicates the importance of social capital. Collaboration among farmers, government agencies and other relevant stakeholders is crucial for developing and implementing strategies to mitigate the impacts of climate change.

Climate change alters suitable habitats for different wild animals and marine species because of the decline in fresh water and drying up of some seasonal pools. The findings made by the researchers on environmental degradation of the riparian ecosystems concurred with Kupika and Nhamo (2016), Mwera, Kupika, & Moyo (2021), and Chanza and Musakwa (2022) who found out that tiger fish vanished in Mwenezi River because of poor quality of water. When agricultural livelihoods failed to put food on the table, people in Matande communal lands engaged in gold panning along Runde River. Inhabitants are encouraged to live in harmony with the mother earth as spelt in the United Nations Sustainable Development Goals 2016 to improve on household food security by deriving complementary food from the riparian ecosystem.

Livestock increases recorded despite the changing climate interestingly, the increase in livestock points to a nexus with food insecurity. Livestock diseases often erupted in the area posing as the main challenge affecting the sale of cattle, which resulted in the quarantine and prohibition of the sale of cattle through formal systems. Similar findings were recorded by Paenda et al. (2020) in the Maranda area of Mwenezi District. Sirdey and Lallau (2020) and Gazaw et al. (2020) elaborated that because of the dynamic nature and complex nature of livestock markets, small-scale farmers were struggling to sell their livestock for their well-being. In Zimbabwe, the Cold Storage Commission (CSC) that used to control trade in the livestock industry now defunct (Paenda et al. 2020; Chingarande et al. 2020). There is need for an improved formal market for the livestock. Small-scale farmers were in the habit of lending cattle to relatives as a way of spreading risk. Tambo et al. (2021) and Nyahunda and Tirivangasi (2021) hold the perception that cattle lending can lead to mal-adaptation, because of the universality of climate change, animals may be trapped with the same disaster resulting in deadly consequences. However, increases in the number of livestock despite the changing climate are testimonial to the fact that government should focus on being livestock centric in diversification coupled with improved formal markets of livestock.

As households were threatened by climate change, the research shows that they deployed a number of copying strategies. The SLF recognises the importance of human skills, knowledge and capabilities in livelihood strategies. Climate change and its associated challenges, such as unpredictable rainfall patterns and pests, require farmers to possess adaptive agricultural techniques and pest management skills. The inhabitants of Matande communal lands responded to hunger by reducing the number of meals uptake per day in line with postulations by Gukurume (2013) and Zvomuya and Mundau (2021). The Zim VAC (2022) attested that approximately 66% of households in Mwenezi district are likely to be food insecure between October 2022 to December 2022. However, such adaptive interventions result in mal-adaptation such as malnutrition and stunting among children (Zim VAC 2022). The researchers also observed that critical community infrastructure was damaged by extreme weather events. Mandimo dam in Matande communal lands was damaged by Cyclone Eline in 2000 and was yet to be repaired.

Poverty among locals precipitated mal-adaptation practices. Filho and Nalau (2018) viewed mal-adaptation practices as unintended negative consequences of climate change adaptation practices. The cutting down of trees for traditional fencing of household gardens, homestead and fields with thorn bushes and poles from indigenous trees were causing climate change through environmental degradation. Sachs (1993:10) argued that ‘Environmental degradation has been discovered to be a worldwide condition of poverty.’ Mataruse, Nyikahadzoi and Fallot (2021) extrapolated that climate change adaptation strategies that have immediate benefits often lead to vulnerability in the future. Everything being said, climate change is now a reality (Chanza 2018); therefore, there is need to invest in adaptation panacea to food insecurity in dry regions.

## Conclusion and recommendations

The study conducted in Matande communal lands, Mwenezi district, Zimbabwe, provides valuable insights into the impacts of climate change on household food security. The findings highlight the challenges faced by the vulnerable populations in the area, including reduced crop yields, changes in food consumption patterns, increased pests and diseases affecting crops and livestock, lack of livestock markets and biodiversity loss. These findings call for urgent actions to enhance community resilience and address the adverse effects of climate change.

To mitigate the impacts of climate change and improve household food security in Matande communal lands, the following recommendations are proposed:

- *Cultivation of drought-tolerant crops*: Promote the adoption and cultivation of drought-tolerant crop varieties that are better suited to withstand the challenges of erratic rainfall patterns and prolonged droughts. This can help ensure more stable crop yields and enhance food security in the face of climate uncertainties.
- *Tapping underground water sources*: explore and utilise underground water sources for irrigation and other purposes to supplement existing water bodies. This will alleviate the pressure on limited water resources, especially in arid regions, and prevent their premature drying, enabling sustained agricultural production and livelihoods.
- *Livestock-centric diversification*: Encourage a livestock-centric approach to diversification in drought-prone areas. This can involve promoting sustainable livestock-rearing practices, improving livestock management and disease control, and facilitating access to formal livestock markets. Enhancing livestock-based livelihoods can provide alternative sources of income and food security for communities.
- *Strengthening climate resilience programmes*: Implement and scale-up climate resilience programmes that equip farmers with knowledge, skills and resources to build resilience in the face of climate change impacts. These programmes should focus on sustainable farming practices, climate-smart agriculture and adaptive strategies tailored to the local context.
- *Enhancing formal livestock markets*: Improve the functioning of formal livestock markets by addressing market inefficiencies, providing market information and infrastructure, and supporting fair trade practices. This will enable small-scale farmers to sell their livestock at fair prices, improving their economic opportunities and food security.
- *Introduction of climate tax to finance and repair damaged critical infrastructure*: The fund helps to mobilise resources to finance adaptation, mitigation and resilience programmes rather than waiting for external interventions. Some of the community dams destroyed by cyclones in the early 2000 were not repaired because of the lack of funding by the government.

Addressing the impacts of climate change on household food security requires a holistic approach, involving collaboration between government agencies, local communities and relevant stakeholders. It is essential to prioritise sustainable practices, build adaptive capacities and promote resilience at individual, community and institutional levels. By implementing these recommendations, Matande communal lands can work towards a more sustainable and food-secure future, even in the face of a changing climate.

### Areas for further studies

- The opportunities presented by the impacts of climate change as they may present opportunities of innovation and investment.
- The possibility of passing the management of livestock and related infrastructure from government to its owners.
